# Past, Present, and Future of Gonadotropin Use in Controlled Ovarian Stimulation During Assisted Reproductive Techniques

**DOI:** 10.7759/cureus.15663

**Published:** 2021-06-15

**Authors:** Anastasia Prodromidou, Elli Anagnostou, Depy Mavrogianni, Emmanouela Liokari, Evangelia Dimitroulia, Petros Drakakis, Dimitrios Loutradis

**Affiliations:** 1 1st Department of Obstetrics and Gynecology, “Alexandra” General Hospital, National and Kapodistrian University of Athens, Medical School, Athens, GRC; 2 School of Medicine, National and Kapodistrian University of Athens, Athens, GRC; 3 In Vitro Fertilization, Fertility Institute, Athens, GRC; 4 Department of Microbiology, Biopathology Unit, Evgenidion Hospital, University of Athens, Medical School, Athens, GRC

**Keywords:** assisted reproductive technology, controlled ovarian stimulation, gonadotropins, human chorionic gonadotropin, human menopausal gonadotropin

## Abstract

A variety of protocols have evaluated the use of several forms of gonadotropins in controlled ovarian stimulation (COS). We aim to review the evolving trends on the use of gonadotropins human chorionic gonadotropin (hCG), follicle-stimulating hormone (FSH) and luteinizing hormone (LH) over time and their combinations in COS for patients who undergo assisted reproductive techniques (ART) protocols. A meticulous search of three electronic databases was performed for articles published in the field up to September 2020. The administration of hCG seems a promising alternative to conventional modalities for COS related to the enhancement of LH activity. The use of gonadotropins was associated with significantly elevated pregnancy rates that ranged from 20.8% to 46.2%. However, the currently available outcomes with regards to oocytes retrieved, number of embryos are still conflicting. A potential beneficial effect was observed by the majority of the studies in terms of the number of embryos and implantation rates, which is, however, highly affected by the type of protocol used (gonadotropin-releasing hormone [GnRH] agonist or antagonist). Further studies are warranted to elucidate the exact pathways of action of gonadotropins in controlled ovarian stimulation to attain the optimal effect.

## Introduction and background

Gonadotropins are hormones enacting a critical role in follicular maturation and in the production and release of ovarian steroidal hormones [1]. Follicle-stimulating hormone (FSH) and luteinizing hormone (LH) are both secreted by the pituitary gland under the stimulation of the gonadotropin-releasing hormone (GnRH), secreted by the hypothalamus [2]. They are responsible for the initial recruitment, growth, selection, and dominance of ovarian follicles as well as for maturation and ovulation, respectively [2]. Both gonadotropins act synergically and have been used in various forms for hormonal controlled ovarian stimulation (COS) in assisted reproductive technology (ART) to increase follicle count. Additionally, human chorionic gonadotropin (hCG) is a hormone that presents biochemical similarities with LH, binds to the same receptor as LH, and therefore, it has also been used in ART [2, 3]. Despite their similarities, LH and hCG are two distinct hormones and activate different affairs through mechanisms that can vary significantly between the two molecules [3]. Additionally, human menopausal gonadotropin (HMG) was first introduced in 1950, and since then, many HMG preparations have been produced, containing different proportions of FSH and LH, aiming at minimizing the LH proportion with the development in modern immunopurification techniques [4].

A variety of protocols have evaluated the use of several forms of gonadotropins either of human purified urine or of artificial recombinant origin [5]. The urine analogues include HMG, urinary FSH (uFSH) and urinary hCG (uhCG). On the other hand, the recombinant regimens that have been applied are recombinant FSH (rFSH), recombinant LH (rLH), and recombinant hCG (rhCG). Selection of the most appropriate regimen among uFSH and rFSH still remains challenging with the currently available literature reporting no significant differences in the number of oocytes retrieved or pregnancy rates among the two types of FSH [5, 6]. The aforementioned gonadotropin preparations have been administered to support COS in infertile women who undergo ART protocols [7]. Hence, no consensus is available on the optimal regimens and preparations [7]. Several studies support the addition of the activity of LH-related regimens to FSH in COS. LH, hCG, and HMG and various combinations of them have been administered with or without FSH for COS [5]. However, the source of LH remains a matter of controversy as well as the exact dosage and route of administration in patients receiving assisted reproductive technologies. More specifically, a recent systematic review on the potential difference of the dose of LH that is contained in HMG and regimens with rFSH plus rLH, both of which can be administered for controlled ovarian stimulation, showed no difference in the examined parameters, including the number of oocytes retrieved, mature oocytes rates, and pregnancy and birth rates among the two protocols [8].

The aim of the present study is to review and analyse the evolving trends on the use of gonadotropins over time and their combinations in COS for patients who undergo ART protocols with special consideration to the emerging role of hCG, LH, and hMG as well as the comparison among them.

## Review

Methods

A thorough search of the currently available literature was performed using three electronic databases: PubMed (1966-2020), Google Scholar (2004-2020), and Scopus (2004-2020). Articles published up to September 2020 were evaluated from the aforementioned databases along with the references of the eligible articles which were retrieved in full text. Studies that report on the use of the gonadotropins LH, HMG, and hCG in various combinations and dosages were evaluated. The following keywords were utilized: “controlled ovarian stimulation”, “gonadotropins”, “human Chorionic Gonadotropin”, “Human menopausal gonadotropin”, “Luteinizing hormone”. Prospective and retrospective original articles (comparative and non-comparative), case reports, and reviews that were written in the English language were assessed and critically appraised. The assessed outcomes included the ART variables and pregnancy outcomes such as gonadotropin doses, the number of retrieved and mature oocytes, fertilization rates, number of transferred embryos, and embryo quality as well as pregnancy and live birth rates.

Results

Efficacy of hCG in COS

The administration of hCG seems promising in COS due to the enhancement of LH activity and has been extensively studied. Two studies reported outcomes with regards to the efficacy of administration of hCG on the in vitro fertilization (IVF)/ intracytoplasmic sperm injection (ICSI) protocols during COS [9, 10]. Their main characteristics and outcomes are presented in Table 1 and Table 2, respectively. 

**Table 1:** Study and patient characteristics rFSH: recombinant-follicle-stimulating hormone, hCG: human chorionic gonadotropin, HMG: human menopausal gonadotropin, GnRH: gonadotropin-releasing hormone, LH: luteinizing hormone, E2: estradiol, RS: retrospective, RCT: randomized controlled trial, SS: statistical significant (p<0.05), NS: non-significant, N/A: not available. Continuous variables were presented as mean±SD except ^a^median (range).

Year; Author	Study period	Type of study	Inclusion criteria	Protocol/ Drug 1	hCG/HMG dose	Patient No	Age (years)	Previous failed attempts	Peak E2
rFSH+hCG versus rFSH alone									
2016; Partsinevelos [9]	07/2012-06/2014	RS	Premenopausal; years ≤48; short GnRHag protocol; normal hormone profile; menstrual cycle of 21-35 days; both ovaries intact	short GnRH agonist Buserelin acetate at day 2 (0.5mg)/ rFSH at day 3 200IU	hCG 100 IU/day on day 3 until triggering	141 vs 124	37.02±4.63 vs 33.99±3.73	1.596±1.454 vs 0.764±0.950	1850.07±1331.19 vs 2152.76±1104.26 (pg/ml)
2009; Beretsos [10]	2008	RCT	Premenopausal; age 25-40 years; normal hormone profile; no ovulation induction or any other treatment for at least 3 months; one previous unsuccessful ICSI	Long GnRH agonist/ rFSH 200IU/day	hCG 200 IU/day pre-treatment for 7 days when ovarian suppression has occurred	19 vs 27	34±4 vs 33±4	1±1 vs 1±0	2125±1190 vs 1643.5±800.2 (pg/ml) SS
rFSH+HMG versus rFSH alone									
2005; Drakakis [11]	2008	RCT	N/A	GnRH agonist/ rFSH 200IU/day for the first 4 days	HMG 1 amp (75IU FSH+ 75IU LH) for 4 days	24 vs 22	32.4±3.1 vs 33±3.7	N/A	1782±1028.4 vs 1638.1±1001.1 (mIU/ml) NS
2003; Loutradis [12]	2002	RS	Previous IVF attempts with long protocol (2-6 times); age ≥37 years	Short GnRH agonist/ rFSH 200IU on day 3	HMG 1 amp for 4 days	98 vs 106	38.09± 5.3 vs 37.33±3.78 NS	2.114±1.243 vs 2.7±1.34 NS	905.9±162 vs 933.8±158 (pg/ml) NS
GnRH agonist+HMG+hCG vs GnRH antagonist+HMG+hCG									
2019; Theofanakis [13]	2014-2016	RCT	Age 32-47 years; no uterine or ovarian anomalies; normal hormonal profile; menstrual cycle of 21-35 days; both ovaries intact	Short agonist	GnRH antag (ganirelix) on day 5+ HMG 200 IU/day on day 3+ hCG 100IU/day on day 2 vs GnRH agon (buserelin) 0.5mg on day 2+ hMG 200 IU/day on day 3+ hCG 100IU	116 vs 124	39.5±3.3 vs 41.4±3.4 SS	N/A	N/A
hCG vs LH									
2009; Drakakis [14]	01/2007 vs 12/2007	RCT	Age 36-42 years; BMI≤32; menstrual cycle of 21-35 days; normal FSH; normal uterine cavity	GnRH agonist/ rFSH 200IU/day on day 3	hCG 200IU for the first 5 days vs LH 200IU for the first 5 days	58 vs 56	36.4±4.2 vs 37.3±1.8 NS	2-6	1888(1119-2118) vs 720 (530-1825)^a^ (pg/ml) SS

**Table 2:** Main outcomes rFSH: recombinant-follicle-stimulating hormone, hCG: human chorionic gonadotropin, HMG: human menopausal gonadotropin, GnRH: gonadotropin-releasing hormone, SS: statistical significant (p<0.05), NS: non-significant, N/A: not available. Continuous variables were presented as mean±SD except ^a^median.

Year; Author	Oocytes retrieved	Mature oocytes	Fertilization rate (%)	No of transferred embryos	Embryo quality	Pregnancy rate (%)	Live birth rate (%)
rFSH+hCG versus rFSH alone							
2016; Partsinevelos [9]	6.31±2.68 vs 8.85±2.71 SS	5.738±2.37 vs 7.323±2.69 SS	81.7±0.141 vs 89.4±0.113 SS	N/A	3.355± 0.575 vs 2.871±0.382 SS	39.7 vs 26.6 N/A	N/A
2009; Beretsos [10]	8±2 vs 7±3 NS	78.9%±18% vs 66.7%±17% NS	85±15 vs 87.5±12.5 NS	N/A	85.3% vs 47.6% NS (>1 Grade 3 embryos)	46.2 vs 31.8 SS	N/A
rFSH+HMG versus rFSH alone							
2005; Drakakis [11]	12.7±6.4 vs 11.8±4.4 NS	10.7±3.4 vs 7.3±2.9 SS	7.9±3 vs 6.5±1.8 SS	7.7±3.1 vs 6±2.8 SS	N/A	20.8 vs 27.8 NS	N/A
2003; Loutradis [12]	7.8±2.7 vs 6.8±3.6 NS	71.1% vs 72.3% NS	71% vs 72.2% NS	4.4±2.1 vs 3.7±3 NS	37.8% vs 14.6 (NS) grade 4; 50.5% vs 59.7% (SS) grade3	26.5 vs 23.6	22.4 vs 20.5
GnRHagonistt+HMG+hCG vs GnRHantagonist+HMG+hCG							
2019; Theofanakis [13]	6.2±6 vs 2.2±2 SS	N/A	N/A	0.9±1 vs 1.4±1 SS	N/A	26.7 vs 12.1 SS	N/A
hCG vs LH							
2009; Drakakis [14]	6 vs 3^a^	75% vs 66.7% NS	71.4% vs 66.7% NS	2.4±0.4 vs 2.5±0.4 NS	N/A	27.6 vs 10.7 SS	N/A

A total of 311 patients were included. Among them, 160 received hCG in addition to rFSH (hCG group) based on each study’s protocol, whereas the remaining 151 patients received rFSH alone (control group) as shown in Table 1. Table 2 presents the main outcomes of those studies. Specifically, despite the fact that no difference was detected by Beretsos et al. [10] in terms of oocytes retrieved, Partinevelos et al. [9] found significantly increased mean number of retrieved oocytes in the control group (p<0.001). The same was also observed concerning the number of mature oocytes as well as the fertilization rates. The aforementioned differences can be attributed to the variances in the design of the protocol of each study. Fertilization rates tended to be higher in the control group but statistical significance was observed only in the study by Partsinevelos et al. [9]. However, in both studies, improved embryo quality levels were reported as well as significantly increased pregnancy rates in women of the hCG group. Consequently, one can hypothesize that the increase in pregnancy rates in the hCG group can be attributed to the higher quality of embryos and is more influenced by the quality of the embryos rather than the number of mature oocytes which was lower in that group. The improvement in the quality of embryos can be attributed to the effect of hCG in inducing the immediate production of androgen from theca cells, which convert into estrogen in granulosa cells by the aromatization process [2]. However, the respective live birth rates were not available.

Outcomes after hCG administration are conflicting based on the findings of the aforementioned studies whilst the currently available literature is also controversial. A recent meta-analysis by Santi et al. that examined the efficacy of addition of hCG in FSH during IVF/ICSI protocols compared to FSH alone, revealed no difference in the number of oocytes retrieved (seven studies included, 948 patients, p=0.850), in the mean number of mature oocytes (five studies included, 352 patients, p=0.730), in the number of embryos (seven studies included, 918 patients, p=0.770), in implantation and pregnancy rates (five studies included, 749 patients, p=0.590 and eight studies, 968 patients, p=0.750, respectively), and in live birth rates (eight studies included, p=0.750) [15]. A meta-analysis by Checa et al. supports the use of hCG in the early or late follicular phase as an efficient alternative to conventional modalities for COS [16]. Specifically, the administration of hCG was found to be associated with improved clinical pregnancy rates when administered in late follicular phase (risk ratio [RR]=1.32, 95% CI, 1.06 to 1.64, p=0.01). Additionally, hCG administration in late follicular phase was related to a significant reduction in the administered doses of FSH (mean difference [MD] -514.68 95% CI -672.86 to -356.51, p<0.00001). This was not observed in the study by Partsinevelos et al., who did not report a difference in mean FSH doses among patients who received hCG and controls (2702.51±1034.62 versus 2692.92±1053.99, p=0.941) [9]. Nonetheless, meta-analysis revealed no difference in live birth rates among the compared groups (RR=1.42, 95% CI 0.97 to 2.08, p=0.07) [16].

The impact of hCG has also been investigated in patients of various age groups. In that setting, Partsinevelos et al. reported significantly improved pregnancy rates in patients who received pre-treatment with hCG additionally to FSH compared to FSH alone in the subgroup of patients aged ≥35 years (=0.002) [9]. This is in accordance with respective studies in the field indicating a beneficial effect of addition of low dose hCG to FSH during COS concerning mature oocytes, fertilization rates, and number of frozen embryos for patients 40 years or older [17].

Furthermore, a beneficial effect of adding rLH to FSH in COS has been recorded in women who have been evaluated as poor responders or those who are >35 years in terms of increased number of oocytes retrieved, implantation, and clinical pregnancy rates [18, 19]. The beneficial effect of additional LH administration did not differ when patients of advanced reproductive age (>35years) were compared to younger ones according to the meta-analysis by Lehert et al [19]. Comparable outcomes have been also reported with the combination of hCG with FSH during COS.

Efficacy of hCG Versus LH

The administration of various gonadotropins combinations has also been evaluated along with their efficacy in different groups of patients. Table 1 depicts the main study and patient characteristics of the trial derived from our institution on comparison of hCG and LH, while Tables 2 shows the main outcomes of the study. A randomized trial by Drakakis et al., which enrolled 114 patients who underwent IVF through a GnRH agonist protocol, compared the 58 patients who received 200IU hCG for the first five days in addition to rFSH versus the other 56 patients who received 200IU rLH for the first five days in addition to rFSH during COS (Tables 1) [14]. According to the findings of this study, the number of oocytes retrieved was significantly higher in the hCG group (p<0.001) (Table 2). The same was also observed in terms of clinical pregnancy rates (p=0.022). The administered FSH dose per patient in the hCG group was significantly lower compared to the rLH group (p<0.001). On the other hand, no difference was noted in the proportion of mature oocytes, fertilized oocytes, and the number of patients with >8 mm endometrium thickness (p=0.752, p=0.317, and p=0.190, respectively) as well as in the implantation rates (p=0.125) and the incidence of moderate ovarian hyperstimulation syndrome (OHSS). The levels of complementary DNA (cDNA) of the LH/hCG receptor were found significantly increased in the lymphocytes of peripheral blood in hCG group compared to LH group (p=0.012). This probably indicates the potential impact of hCG in an improved availability and management of the receptor under the effect of the gonadotropin. Accordingly, elevated levels of cDNA were measured in women who achieved pregnancy compared to those who did not, but statistical significance was not reached (p=0.687).

The reproductive outcomes of the aforementioned study are in contrast with those reported in another conducted Randomized Controlled Trial (RCT) by Alsbjerg et al. that compared administration of rHCG with rLH to FSH from the first day of COS with a one-to-one bioactivity [20]. More specifically, no difference in the number of oocytes retrieved and in live birth rates were identified [20]. Estradiol levels on the day of triggering were additionally measured, which were also not significantly different [20]. The supplementation of LH to FSH has been related to favourable ovarian outcomes based on the hypothesis that LH causes suspension of LH suppression from the ovary compared with the single use of FSH as monotherapy [21]. Nonetheless, a recent Cochrane systematic review showed no difference with regards to live birth rates and incidence of OHSS in patients who received additional rLH to FSH compared to FSH monotherapy in COS [22]. Moreover, no improvement was observed in reproductive outcomes including number of retrieved oocytes, implantation rate, pregnancy rate, and liver birth rates in patients aged ≥35 years who received additional rLH to rFSH compared to those who received FSH alone according to the RCT by Vuong et al. [23]. Comparable outcomes were also reported in the RCT by König et al. for women of advanced reproductive age [24].

Efficacy of HMG in COS

The contribution of adding HMG to FSH during COS is controversial. The outcomes after addition of HMG in COS are reported in the studies by Loutradis et al. [12] and Drakakis et al. [11] who included a total of 250 patients. The main study and patients’ characteristics as well as the outcomes of the aforementioned studies are available in Table 1 and Table 2, respectively. Among them, 122 patients received 1 amp HMG for four days additionally to rFSH (HMG group) while the remaining 128 patients received rFSH alone (control group) (Table 1). In both studies GnRH agonist protocols, either long or short, were followed. No difference was observed in the number of oocytes retrieved and the rates of mature oocytes (Table 2). Despite the fact that Loutradis et al. did not show any differences in fertilization rates and number of transferred embryos between the two groups, Drakakis et al. detected significantly elevated fertilization rates and mean number of embryos transferred in HMG group compared to control (p<0.001). Loutradis et al. documented significantly improved quality of embryos in the group of HMG addition. The same authors reported pregnancy rates of 27% for patients in the HMG group compared to 24% for those in the control group, but significance was barely reached (p=0.05). Finally, live birth rates did not differ among the two groups [12].

According to the meta-analysis by Santi et al. for patients who received GnRH agonist protocol no difference was observed in terms of mean number of oocytes retrieved (p=0.11) and implantation rates (p=0.06) among patients who received HMG compared to those who received FSH alone during COS. Meanwhile, mean number of embryos (p<0.001) and pregnancy rates (p=0.030) were significantly elevated in patients in the GnRH agonist protocol who received HMG. On the contrary, for patients in GnRH antagonist protocols the mean number of embryos (p=0.86) as well as pregnancy rates (p=0.370) were not different among the two groups. When the overall effect irrespective of the protocol was calculated, the number of retrieved oocytes was significantly elevated in the FSH control group (p<0.001), but the HMG group presented significantly elevated number of embryos (p<0.001) and implantation rates (p=0.03). However, pregnancy rates did not differ among the two groups (p=0.1). The discrepancy in the outcomes between the two protocols (GnRH agonist and GnRH antagonist) was attributed by the authors to the difference in the mechanism of each analog indicating the potential beneficial effect on the use of HMG in GnRH antagonist protocols in selected patients where mild stimulation is expected [15]. A recently published RCT that compared the efficacy of COS with highly purified (HP)-HMG plus rFSH versus rFSH alone in long GnRH agonist protocols showed that although the rFSH group was found with significantly increased retrieved oocytes, fertilization rates were higher in the HMG group whereas implantation and pregnancy rates were not different among the two groups [7]. Another systematic review by Levi Setti et al. that presented the outcomes of studies comparing the administration of rFSH with HMG during COS observed that in the majority of included studies (10 out of 13), the number of retrieved oocytes was significantly higher in the rFSH group and the cost for both therapies was comparable [25]. Additionally, in the RCT by Figen Turkcapar et al., a decreased proportion of retrieved oocytes was noted in patients who received HMG compared to rFSH during COS (p=0.002) in patients with polycystic ovarian syndrome, while no difference was demonstrated in fertilization rates, number of embryos transferred, and pregnancy rates among the two groups [26]. A proportion of 11.9% of patients were diagnosed with OHSS in the rFSH group whereas no patient from the HMG group presented with OHSS [26].

Efficacy of hCG and HMG in the Type of Protocol

The impact of administration of low dose hCG and HMG in the type of protocol has been evaluated in the study by Theofanakis et al. (Table 1) [13]. More specifically, the aforementioned regimens were used for COS in patients that underwent GnRH antagonist protocol (124 patients) versus short GnRH agonist protocol (116 patients) (Table 2) [13]. The authors reported significantly improved outcomes with regards to the number of oocytes retrieved and pregnancy rates in patients from the GnRH agonist protocol who underwent COS with HMG and hCG. Accordingly, subgroup analysis in patients aged over 40 years showed significantly higher rates of oocytes and follicles as well as pregnancy rates in patients from the GnRH agonist group. This study indicated the potential superiority of GnRH agonist protocol during COS compared to administration of GnRH antagonist protocol. This can be attributed to the endometrial receptivity as reported by Orvieto et al. who observed that patients who underwent GnRH agonist protocols had significantly more thick endometrium, which also reflected in higher pregnancy rates when compared to GnRH antagonist protocol during controlled ovarian hyperstimulation (COH) [27]. Similar outcomes were also reported in a recent analysis by Geng et al. [28]. The potential adverse effect of GnRH antagonist protocols is also reflected by Kolanska et al who also found decreased pregnancy rates and live birth rates in patients with infertility due to endometriosis, compared to those who underwent GnRH agonist protocols [29].

Discussion

The objective of the present study was to present a comprehensive report on the use of gonadotropins in COS for patients who undergo ART protocols during the past 30 years focusing in particular on the role of hCG, LH, and HMG and the comparison among them. The findings of the present study indicate that the administration of hCG seems a promising alternative to conventional modalities for COS related to enhancement of the LH activity. However, the currently available evidence with regards to the number of oocytes retrieved, the number of embryos and the clinical pregnancy rates are still conflicting. A potential beneficial effect was observed by the majority of the studies with the use of gonadotropins in terms of the number of embryos and implantation rates which is however highly affected by the type of protocol used (GnRH agonist or antagonist).

Controlled ovarian stimulation during IVF may result in significantly variable ovarian response among women, with adverse outcomes in patients of advanced age or those with history of previous failed attempts and poor responders. Following the systematic evaluation of the LH/hCG receptor pathways of action, knowledge with regards to the potential effects of hCG has been extended during the past decades [30]. It has been proved that irrespective of FSH, the use of hCG in low doses, resulted in maturation of larger ovarian follicles with granulosa cell LH/hCG receptors and thus representing an effective ovarian induction tool. [14]. Specifically, the induction of androgen production by theca cells results in a subsequent increment in aromatization rate and transformation of androgens to estrogens by granulosa cells [31]. It seems that not only has hCG a favorable effect in the induction of ovulation but the gonadotropin has also an additional effect on the immune system, the receptivity of endometrium, and, most importantly, on the follicle microenvironment and nuclear maturation of the oocyte, which are all considered of paramount importance in human reproduction [2, 32]. Furthermore, the addition of hCG impacts the hormonal follicular traits in favor of a more androgenic environment by stimulating the levels of intrafollicular androgen and estradiol [32]. Consequently, this androgenic environment is related to improved embryo quality [32]. Moreover, an experimental study on in vitro oocyte maturation in mice has demonstrated that compared to recombinant LH, hCG resulted a significant elevation in higher maturation rates in cultures of mouse germinal vesicle oocytes [33]. This could be probably explained by the presence of the LH/hCG receptor on oocytes at this stage of development and the variances in the half time of hCG and LH [33].

The clinical observations that emerge from a 30-year experience with regards to the use of hCG in COS, along with the outcomes of randomized controlled trials and recent meta-analysis in this field, suggest the beneficial “hCG effect” in COS through the application various protocols (long GnRH-agonist down-regulation protocol, GnRH short, or GnRH antagonist) with special consideration to the associated impact in women with previous ART failures or in those of advanced reproductive age.

The meta-analysis by Checa et al. evaluated the potential beneficial effect of hCG for the induction of follicular development in COS from a total of 11 randomized controlled trials that included 1068 patients [16]. Specifically, the authors demonstrated the favorable outcomes of hCG in the increment of clinical pregnancy rates only when administered in late follicular phase (data from five studies) while no difference was observed in administration at early follicular phase (data from three studies) [16]. Additionally, they presented comparable efficacy to traditional COS strategies with the additional advantage of reducing the doses of FSH in women who received hCG [16]. Finally, significantly elevated number of mature oocytes were retrieved in patients who received FSH alone compared to those who received late follicular phase hCG (10.6 ± 0.5 versus 10.3 ± 0.5, p<0.0001) [16]. Comparable outcomes were presented in the meta-analysis by Santi et al. which demonstrated that FSH alone resulted in significantly elevated oocyte numbers whereas on the other hand, a significant improvement in the number of embryos and the implantation rates were observed with the administration of HMG [15]. They showed that adding rLH to FSH led to increased pregnancy rates probably by the reported reduce in the dose of FSH. In GnRH agonist protocols, FSH + LH and HMG treatment both resulted in improved pregnancy rates while FSH + hCG or HMG alone were found with equal effectiveness in terms of pregnancy rates when compared to FSH alone. However, the inclusion and exclusion criteria of each individual study showed significant diversity which makes the comprehensive interpretation of these outcomes equivocal. A significant discrepancy exists among the published data; initially, the significant heterogeneity of the reported outcomes of the included studies consists a critical limitation of those two meta-analyses. A variation is observed in the methodological assessment of the outcomes of each meta-analysis, meanwhile, it is obvious that both provided different interpretations of the results and thus making the decision on the appropriate regimen ambiguous.

Recent advances in genetics, have introduced the concept of tailoring regimen for ovulation induction for each individual patient. As such, each patient's genetic profile is separately evaluated with the intention to predict the response to gonadotropin stimulation. Pharmacokinetics is the study of the relationship between individual gene variants and variable drug effects and represents a promising field with evolving interest which highlights the impact of the variances in DNA sequence on the drug response, efficacy, and adverse events. The most studied genes in the literature with relevant clinical implication are those related to the estrogen pathway and their receptors: FSH, anti-Müllerian hormone (AMH), and LH receptors. Ser680Asn polymorphism of the FSH receptor (FSHR) is the most studies single nucleotide polymporphism (SNP). This polymorphism seems to impact the ovarian response to stimulation which is induced by FSH in IVF cycles since carriers of the Ser/Ser genotype have been proved with resistance in FSH action. This may enable the performance of a diagnostic test which could predict the specific ovarian response and determine the dose of FSH while simultaneously prevent potential complications [34]. With regards to LH receptor (LHR), carriers of splice variants of the receptor and more specifically 735 bp and 621 bp demonstrated improved outcomes [35].

In perspective, the FSHR and LHR genotype should be studied in relation to stimulation by different FSH, rLH, or hCG isoforms and variants. An increasing number of synthetic, recombinant gonadotropins are currently produced and used as equivalent to the natural molecules in ART protocols. However, different gonadotropins preparations with individual isoform composition, physiological functions, and receptor binding properties could contribute in the improvement of the clinical parameters. In this manner, the identification of the molecular mechanisms through which gonadotropins and receptor isoforms and variants modulate the cell signaling will extend the global horizons and provide us with novel, significant therapeutic approaches for assisted reproduction.

## Conclusions

The administration of hCG seems to be a promising alternative to conventional modalities for COS related to enhancement of the LH activity. Nonetheless, the currently available evidence is still controversial in terms of the number of oocytes retrieved, the number of embryos, and the clinical pregnancy rates. Further studies are warranted to elucidate the exact pathways of gonadotropins' action in COS and thus to enhance the effect of these agents. Optimization of ART modalities and achievement of pregnancy represent the primary objectives of the aforementioned strategies.

## References

[1] Filatov M, Khramova Y, Parshina E, Bagaeva T, Semenova M (2017). Influence of gonadotropins on ovarian follicle growth and development in vivo and in vitro. Zygote.

[2] Theofanakis C, Drakakis P, Besharat A, Loutradis D (2017). Human chorionic gonadotropin: the pregnancy hormone and more. Int J Mol Sci.

[3] Choi J, Smitz J (2014). Luteinizing hormone and human chorionic gonadotropin: origins of difference. Mol Cell Endocrinol.

[4] Lunenfeld B, Bilger W, Longobardi S, Alam V, D'Hooghe T, Sunkara SK (2019). The development of gonadotropins for clinical use in the treatment of infertility. Front Endocrinol (Lausanne).

[5] Casarini L, Brigante G, Simoni M, Santi D (2016). Clinical applications of gonadotropins in the female: assisted reproduction and beyond. Prog Mol Biol Transl Sci.

[6] van Wely M, Kwan I, Burt AL, Thomas J, Vail A, Van der Veen F, Al-Inany HG (2011). Recombinant versus urinary gonadotrophin for ovarian stimulation in assisted reproductive technology cycles. Cochrane Database Syst Rev.

[7] Shu L, Xu Q, Meng Q (2019). Clinical outcomes following long GnRHa ovarian stimulation with highly purified human menopausal gonadotropin plus rFSH or rFSH in patients undergoing <i>in vitro</i> fertilization-embryo transfer: a multi-center randomized controlled trial. Ann Transl Med.

[8] Orvieto R (2019). HMG versus recombinant FSH plus recombinant LH in ovarian stimulation for IVF: does the source of LH preparation matter?. Reprod Biomed Online.

[9] Partsinevelos GA, Antonakopoulos N, Kallianidis K, Drakakis P, Anagnostou E, Bletsa R, Loutradis D (2016). Addition of low-dose hCG to rFSH during ovarian stimulation for IVF/ICSI: is it beneficial?. Clin Exp Obstet Gynecol.

[10] Beretsos P, Partsinevelos GA, Arabatzi E (2009). "hCG priming" effect in controlled ovarian stimulation through a long protocol. Reprod Biol Endocrinol.

[11] Drakakis P, Loutradis D, Kallianidis K (2005). Small doses of LH activity are needed early in ovarian stimulation for better quality oocytes in IVF-ET. Eur J Obstet Gynecol Reprod Biol.

[12] Loutradis D, Elsheikh A, Kallianidis K, Drakakis P, Stefanidis K, Milingos S, Michalas S (2004). Results of controlled ovarian stimulation for ART in poor responders according to the short protocol using different gonadotrophins combinations. Arch Gynecol Obstet.

[13] Theofanakis C, Athanasiou V, Liokari E (2019). The impact of HCG in IVF treatment: does it depend on age or on protocol?. J Gynecol Obstet Hum Reprod.

[14] Drakakis P, Loutradis D, Beloukas A (2009). Early hCG addition to rFSH for ovarian stimulation in IVF provides better results and the cDNA copies of the hCG receptor may be an indicator of successful stimulation. Reprod Biol Endocrinol.

[15] Santi D, Casarini L, Alviggi C, Simoni M (2017). Efficacy of follicle-stimulating hormone (FSH) alone, FSH + luteinizing hormone, human menopausal gonadotropin or FSH + human chorionic gonadotropin on assisted reproductive technology outcomes in the "personalized" medicine era: a meta-analysis. Front Endocrinol (Lausanne).

[16] Checa MA, Espinós JJ, Requena A (2012). Efficacy and safety of human chorionic gonadotropin for follicular phase stimulation in assisted reproduction: a systematic review and meta-analysis. Fertil Steril.

[17] Gomaa H, Casper RF, Esfandiari N, Chang P, Bentov Y (2012). Addition of low dose hCG to rFSh benefits older women during ovarian stimulation for IVF. Reprod Biol Endocrinol.

[18] Hill MJ, Levens ED, Levy G, Ryan ME, Csokmay JM, DeCherney AH, Whitcomb BW (2012). The use of recombinant luteinizing hormone in patients undergoing assisted reproductive techniques with advanced reproductive age: a systematic review and meta-analysis. Fertil Steril.

[19] Lehert P, Kolibianakis EM, Venetis CA (2014). Recombinant human follicle-stimulating hormone (r-hFSH) plus recombinant luteinizing hormone versus r-hFSH alone for ovarian stimulation during assisted reproductive technology: systematic review and meta-analysis. Reprod Biol Endocrinol.

[20] Alsbjerg B, Elbaek HO, Laursen RJ, Povlsen BB, Haahr T, Yding Andersen C, Humaidan P (2017). Bio-equivalent doses of recombinant HCG and recombinant LH during ovarian stimulation result in similar oestradiol output: a randomized controlled study. Reprod Biomed Online.

[21] De Placido G, Alviggi C, Perino A (2005). Recombinant human LH supplementation versus recombinant human FSH (rFSH) step-up protocol during controlled ovarian stimulation in normogonadotrophic women with initial inadequate ovarian response to rFSH. A multicentre, prospective, randomized controlled trial. Hum Reprod.

[22] Mochtar MH, Danhof NA, Ayeleke RO, Van der Veen F, van Wely M (2017). Recombinant luteinizing hormone (rLH) and recombinant follicle stimulating hormone (rFSH) for ovarian stimulation in IVF/ICSI cycles. Cochrane Database Syst Rev.

[23] Vuong TN, Phung HT, Ho MT (2015). Recombinant follicle-stimulating hormone and recombinant luteinizing hormone versus recombinant follicle-stimulating hormone alone during GnRH antagonist ovarian stimulation in patients aged ≥35 years: a randomized controlled trial. Hum Reprod.

[24] König TE, van der Houwen LE, Overbeek A (2013). Recombinant LH supplementation to a standard GnRH antagonist protocol in women of 35 years or older undergoing IVF/ICSI: a randomized controlled multicentre study. Hum Reprod.

[25] Levi Setti PE, Alviggi C, Colombo GL (2015). Human recombinant follicle stimulating hormone (rFSH) compared to urinary human menopausal gonadotropin (HMG) for ovarian stimulation in assisted reproduction: a literature review and cost evaluation. J Endocrinol Invest.

[26] Figen Turkcapar A, Seckin B, Onalan G, Ozdener T, Batioglu S (2013). Human menopausal gonadotropin versus recombinant FSH in polycystic ovary syndrome patients undergoing in vitro fertilization. Int J Fertil Steril.

[27] Orvieto R, Meltzer S, Rabinson J, Zohav E, Anteby EY, Nahum R (2008). GnRH agonist versus GnRH antagonist in ovarian stimulation: the role of endometrial receptivity. Fertil Steril.

[28] Geng Y, Xun Y, Hu S, Lai Q, Jin L (2019). GnRH antagonist versus follicular-phase single-dose GnRH agonist protocol in patients of normal ovarian responses during controlled ovarian stimulation. Gynecol Endocrinol.

[29] Kolanska K, Cohen J, Bendifallah S (2017). Pregnancy outcomes after controlled ovarian hyperstimulation in women with endometriosis-associated infertility: GnRH-agonist versus GnRH-antagonist. J Gynecol Obstet Hum Reprod.

[30] Filicori M, Fazleabas AT, Huhtaniemi I, Licht P, Rao ChV, Tesarik J, Zygmunt M (2005). Novel concepts of human chorionic gonadotropin: reproductive system interactions and potential in the management of infertility. Fertil Steril.

[31] Skinner MK, Schmidt M, Savenkova MI, Sadler-Riggleman I, Nilsson EE (2008). Regulation of granulosa and theca cell transcriptomes during ovarian antral follicle development. Mol Reprod Dev.

[32] Thuesen LL, Andersen AN, Loft A, Smitz J (2014). Intrafollicular endocrine milieu after addition of hCG to recombinant FSH during controlled ovarian stimulation for in vitro fertilization. J Clin Endocrinol Metab.

[33] Dinopoulou V, Drakakis P, Kefala S (2016). Effect of recombinant-LH and hCG in the absence of FSH on in vitro maturation (IVM) fertilization and early embryonic development of mouse germinal vesicle (GV)-stage oocytes. Reprod Biol.

[34] Anagnostou E, Mavrogianni D, Theofanakis C (2012). ESR1, ESR2 and FSH receptor gene polymorphisms in combination: a useful genetic tool for the prediction of poor responders. Curr Pharm Biotechnol.

[35] Papamentzelopoulou M, Mavrogianni D, Partsinevelos GA (2012). LH receptor gene expression in cumulus cells in women entering an ART program. J Assist Reprod Genet.

